# Supplementary material for: Comprehensive Lineage Tracing Maps the Landscape of Cell Fate Decisions in Mouse Embryogenesis
Source: Cell. Author manuscript; Available in PMC 2026 Oct 30. (PMC13722109; doi:10.1016/j.cell.2026.09.050)
Supplement: Supplementary file 1 [file NIHMS2214476-supplement-1.pdf]

Supplemental information

Comprehensive Lineage Tracing Maps the Landscape of Cell Fate Decisions in Mouse Embryogenesis

William N. Colgan[1,2,3,4†], Luke W. Koblan[1,2,3,4†**], JoAnne Villagrana[5*], Tien-Chi Jason Hou[5*], Minming Wang[5*], Gokul Gowri[1,2,3,4,6,7], Whitney Chandler[1], Leonardo A. Sepúlveda[2,8,9], Didar Ciftci[2,8,9], Karina Smolyar[1,2,3,4], Kathryn E. Yost[1,2,3,4], Alicia Young[1], Lars Wittler[10], Styliani Markoulaki[1,‡], Kyle M. Loh[11], Xiaowei Zhuang[2,8,9], Nir Yosef[12], Zachary D. Smith[5,10**], Jonathan S. Weissman[1,2,3,4**]

[1]Whitehead Institute for Biomedical Research; Cambridge MA, 02142, USA
[2]Howard Hughes Medical Institute; Chevy Chase MD, 20815, USA
[3]David H. Koch Center for Integrative Cancer Research, MIT, Cambridge, MA 02139, USA
[4]Department of Biology, MIT, Cambridge MA 02139 USA
[5]Department of Genetics, Yale Stem Cell Center, Yale School of Medicine; New Haven CT 06519, USA
[6]Laboratory for Information and Decision Systems, MIT, Cambridge, MA, 02139, USA
[7]Schwarzman College of Computing, MIT, Cambridge, MA 02139, USA
[8]Department of Chemistry and Chemical Biology, Harvard University; Cambridge MA, 02138, USA
[9]Department of Physics, Harvard University; Cambridge MA, 02138, USA
[10]Max Planck Institute of Molecular Genetics, Max Planck Society; Berlin, Germany, 14195
[11]Department of Developmental Biology, Institute for Stem Cell Biology and Regenerative Medicine, Stanford University, Stanford CA, 94305, USA
[12]Department of Systems Immunology, Weizmann Institute of Science; Rehovot, 7610001, Israel
‡Current address: Targeted Mutagenesis Laboratory, Feinberg School of Medicine, Northwestern University; Chicago IL, 60611, USA
†Equal contribution
*Equal contribution
**Correspondence: lkoblan@wi.mit.edu, z.smith@yale.edu, weissman@wi.mit.edu

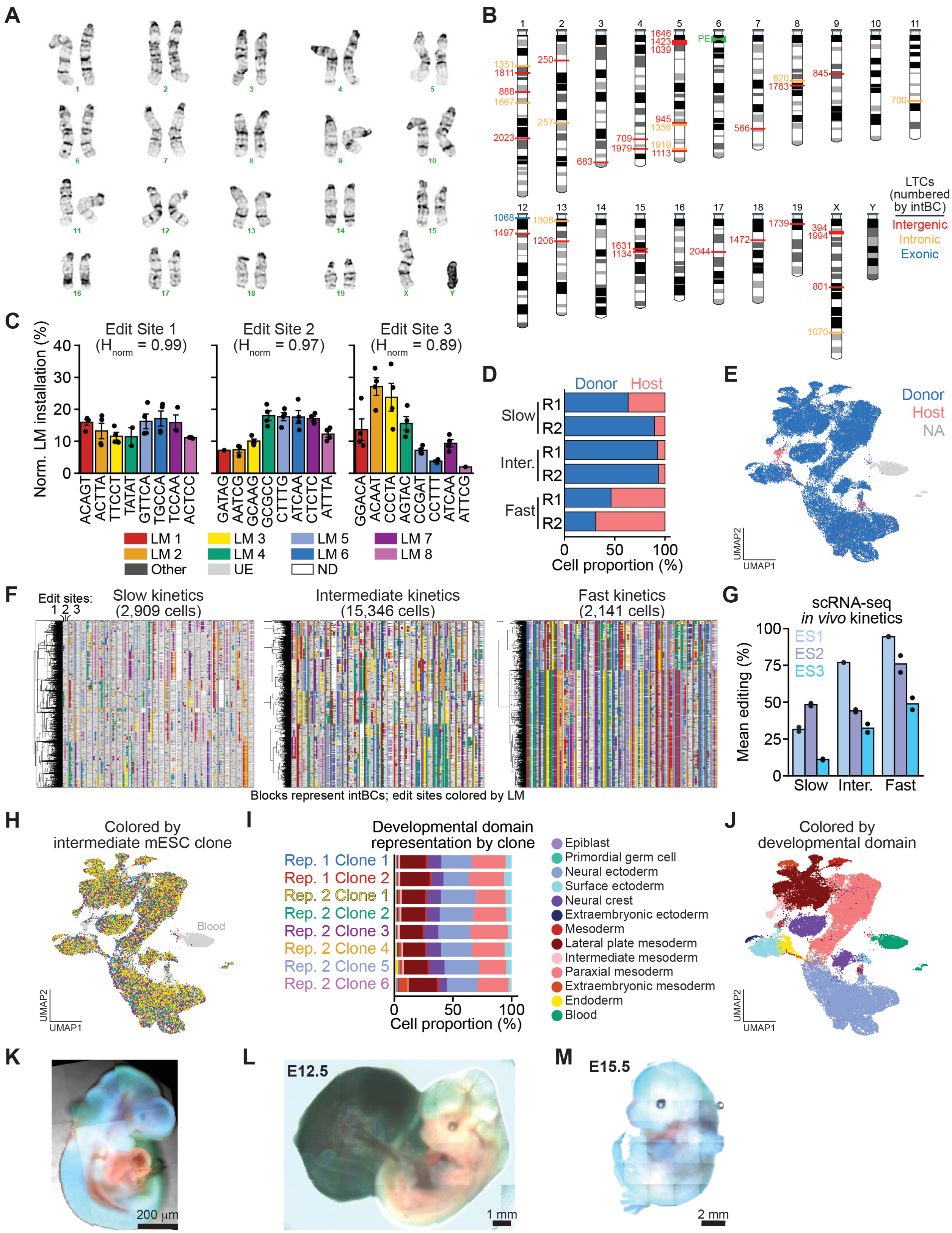

Figure S1. Validation of PEtracer for mouse developmental studies, related to Figure 1.

(**A**) Karyotype analysis of mouse embryonic stem cells harboring a homozygous Rosa26 knock-in of Cre-activatable PEmax-T2A-GFP and 35 unique, genomically integrated lineage tracing cassettes (LTCs). (**B**)

Genomic positions of integrated LTCs numbered by integration barcode (intBC) and colored by genomic context: intergenic (red), intronic (orange), a single exonic integration in *Ncoa1* (blue), and Rosa26 PEmax-T2A-GFP knock-in on chromosome 6 (green). (**C**) Normalized (Norm.) lineage mark (LM) installation efficiencies for all eight LMs at each of the three edit sites (ESs) in intermediate-kinetics E9.5 embryos from bulk sequencing. Bars represent the mean of four biological replicates ± standard error of the mean; normalized entropy (H_{norm}) values provided for each ES. LMs are colored and ordered by their position in the pegArray. (**D**) Proportion of tracing donor cells (blue) and non-tracing host cells (salmon) assessed by single-cell RNA sequencing (scRNA-seq) for two biological replicates of E9.5 embryos generated from slow-, intermediate- (inter.), and fast-kinetics mESC lines. (**E**) UMAP embedding of two intermediate-kinetics E9.5 embryos colored by donor and host cell identity with primitive erythrocytes excluded. (**F**) Cellular phylogenies and character matrices for representative sub-sampled slow-, intermediate-, and fast-kinetics E9.5 embryos. Character matrix blocks represent LTCs with distinct intBCs and are colored by LM identity at ESs 1-3; cell number is indicated for each tree. (**G**) *In vivo* LM installation rates for donor cells from the two E9.5 embryo replicates in (D). (**H**) UMAP embedding from (E) colored by contributing mESC clone. (**I**) Proportions of cell types in E9.5 embryos derived from the mESC clones in (H). (**J**) UMAP embedding from (E) colored by developmental domain. Representative embryos derived from injection of activated PEtracer cells into tetraploidized host morulae at (**K**) E9.5, (**L**) E12.5, and (**M**) E15.5.

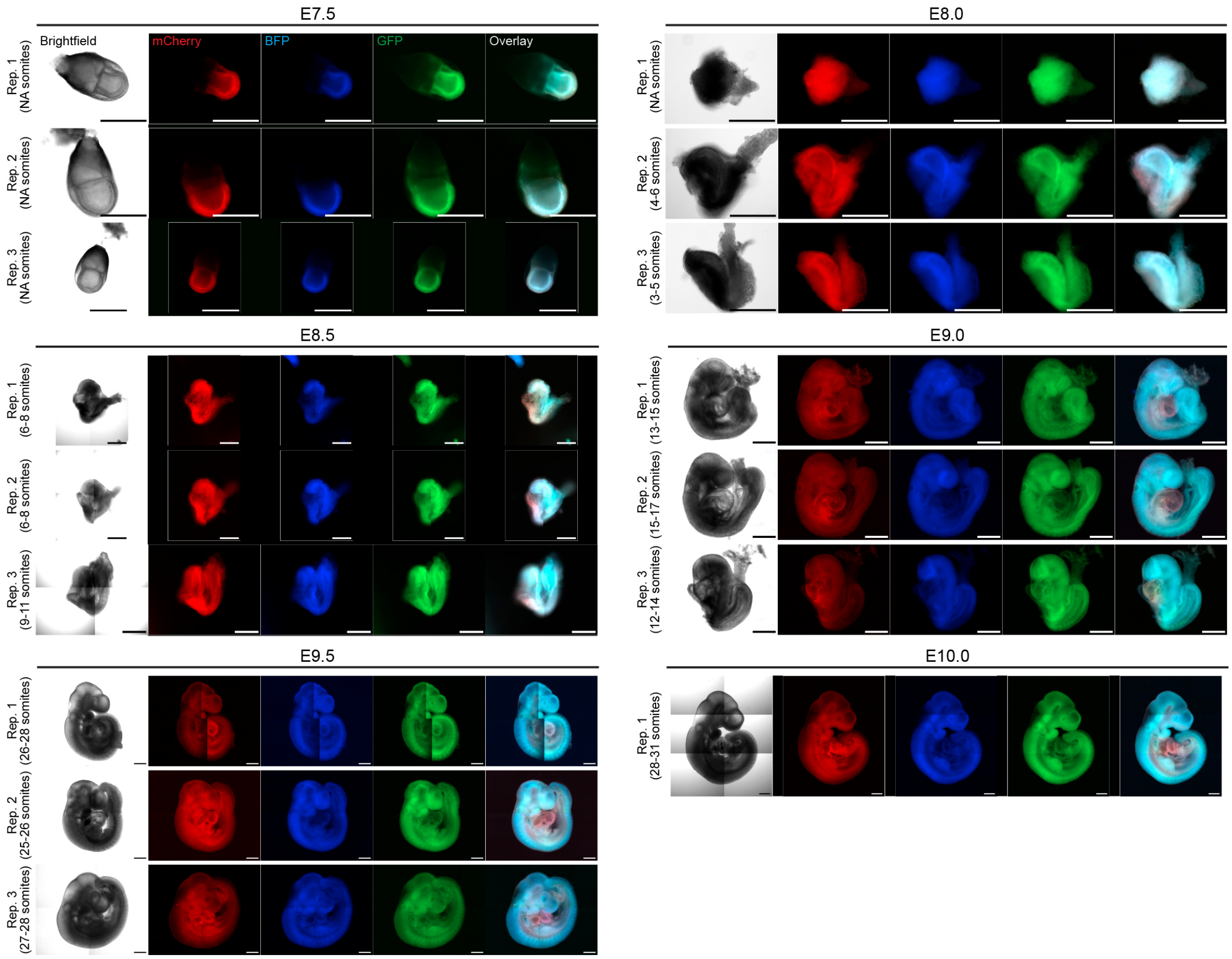

Figure S2. Fluorescent characterization of PEtracer embryos collected across E7.5-E10.0, related to Figure 1.

Representative imaging of PEtracer embryos spanning E7.5 to E10.0 prior to dissociation and sequencing. For each embryo, four channels are shown: brightfield, PEmax-T2A-GFP (green), mCherry-linked LTCs (red), and BFP-linked pegArray (blue), along with an overlay of all fluorescent signals. Embryos are displayed in chronological order of developmental stage to illustrate progressive changes in morphology and reporter expression across gastrulation and early organogenesis. Biological triplicates were collected for each developmental stage except E10.0. All scale bars = 500 μm. Somite counts are indicated for each embryo where determinable (NA, not assessable).

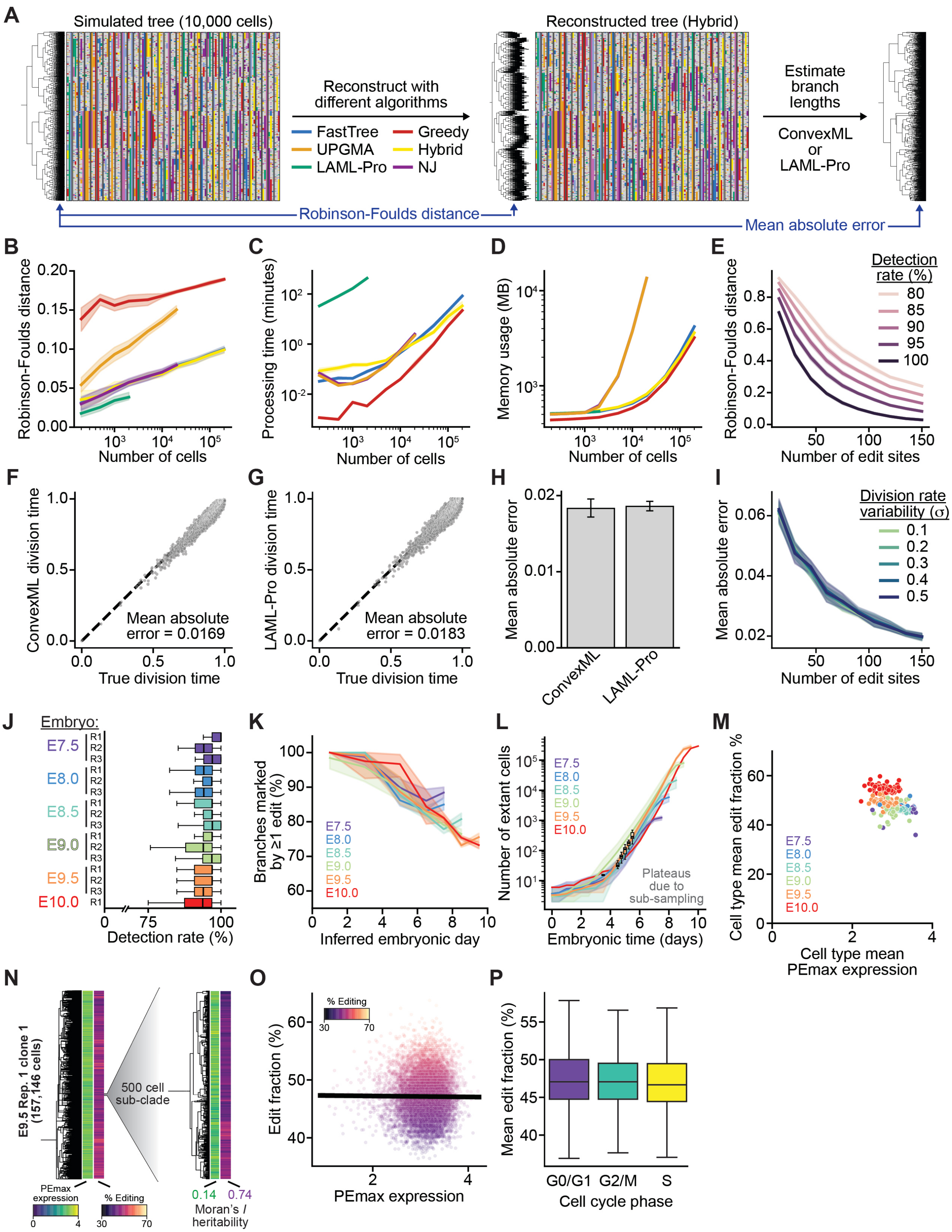

A
Simulated tree (10,000 cells)
Reconstruct with different algorithms
FastTree
UPGMA
LAML-Pro
Greedy
Hybrid
NJ
Reconstructed tree (Hybrid)
Estimate branch lengths
ConvexML or LAML-Pro
Robinson-Foulds distance
Mean absolute error
B
Robinson-Foulds distance
Number of cells
C
Processing time (minutes)
Number of cells
D
Memory usage (MB)
Number of cells
E
Robinson-Foulds distance
Detection rate (%)
80
85
90
95
100
Number of edit sites
F
ConvexML division time
Mean absolute error = 0.0169
True division time
G
LAML-Pro division time
Mean absolute error = 0.0183
True division time
H
Mean absolute error
ConvexML
LAML-Pro
I
Mean absolute error
Division rate variability (σ)
0.1
0.2
0.3
0.4
0.5
Number of edit sites
J
Embryo:
E7.5
E8.0
E8.5
E9.0
E9.5
E10.0
R1
R2
R3
Detection rate (%)
K
Branches marked by ≥1 edit (%)
Inferred embryonic day
L
Number of extant cells
Plateaus due to sub-sampling
Embryonic time (days)
M
Cell type mean edit fraction %
Cell type mean PEmax expression
N
E9.5 Rep. 1 clone 1 (157,146 cells)
500 cell sub-clade
PEmax expression
% Editing
0.14
0.74
Moran's I / heritability
O
% Editing
Edit fraction (%)
PEmax expression
P
Mean edit fraction (%)
G0/G1
G2/M
S
Cell cycle phase

Figure S3. Benchmarking lineage tree reconstruction on simulated data and validation in PEtracer embryos, related to Figure 1.

(**A**) Schematic of the simulation-based benchmarking framework. Ground-truth lineage trees and tracing data were simulated (**Methods**), then reconstructed using six algorithms: FastTree (blue)[1], UPGMA (orange), LAML-Pro (green)[2], Greedy (red)[3], Hybrid (yellow), and Neighbor-Joining (NJ; purple). Reconstruction accuracy was quantified as the normalized Robinson-Foulds (RF) distance between the simulated and reconstructed topologies. Branch lengths were subsequently estimated using either ConvexML[4] or LAML-Pro[2], and division-timing accuracy was quantified as the mean absolute error (MAE) relative to ground truth. (**B**) Normalized RF distance between simulated and reconstructed trees as a function of the number of cells for each reconstruction algorithm. (**C**) Processing time (minutes) and (**D**) peak memory usage (MB) for each algorithm as a function of the number of cells. (**E**) Normalized RF distance for the Hybrid approach as a function of the number of edit sites, colored by LM detection rate. (**F, G**) Inferred versus ground truth division time for each internal node using (**F**) ConvexML (MAE = 0.0169) and (**G**) LAML-Pro (MAE = 0.0183); dashed line indicates y = x. (**H**) MAE of inferred division times for ConvexML and LAML-Pro across 10 replicates. (**I**) MAE of ConvexML-inferred division times as a function of the number of edit sites, colored by division rate variability (σ = 0.1 - 0.5). For (B–E) and (I), lines represent the mean and shaded regions the 95% confidence interval across 10 simulated replicates. (**J**) Lineage mark (LM) detection rate by scRNA-seq across all sampled embryos; R = replicate. (**K**) Mean fraction of branches marked by at least one edit across all sampled embryos, colored by timepoint. (**L**) Mean estimated number of extant cells per embryonic timepoint as a function of inferred time based on branch length estimates. Boxplots indicate published epiblast cell count estimates between E3.5 and E5.5[5]. (**M**) PEmax expression versus mean edit fraction of cell types colored by embryonic timepoint. (**N**) Representative cellular lineage tree for E9.5 R1 clone 1, comprising 157,146 cells with a 500 cell sub-clade also shown. Color bars indicate PEmax expression (left) and percent of edit sites marked with an LM (% editing, right), for each cell. Moran's *I* heritability provided for both PEmax expression (green) and edit fraction (purple). Edit fraction versus (**O**) PEmax expression and (**P**) by cell cycle phase for all cells in E9.5 R1 clone 1.

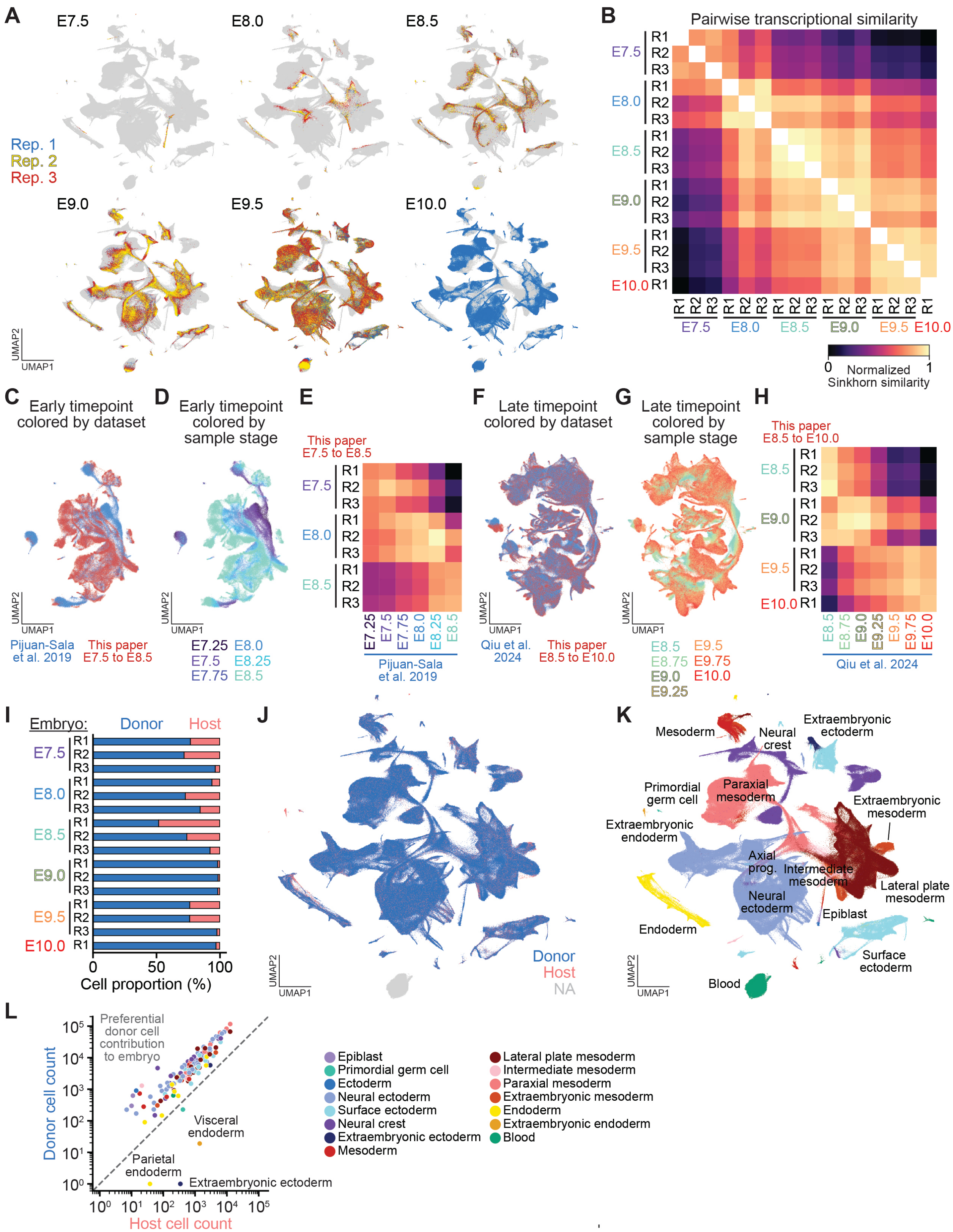

Figure S4. Transcriptional staging and chimerism quantification of PEtracer embryos, related to Figure 2.

(**A**) UMAP embedding of all 1,792,520 cells from 16 embryos spanning E7.5 to E10.0 (see Figure 2A). Cells from each of three biological replicates for the indicated timepoint are colored separately (Rep. 1, blue; Rep. 2, yellow; Rep. 3, red), with cells from the other timepoints shown in gray. (**B**) Heatmap of pairwise transcriptional similarity between all replicates across timepoints, quantified by normalized Sinkhorn similarity. High within-stage similarity and graded between-stage similarity reflect the continuous progression of embryonic development. (**C-E**) Integration of the early timepoints from this study (E7.5-E8.5) with the reference dataset from Pijuan-Sala et al., 2019[6]. (**C**) Joint UMAP colored by dataset of origin (Pijuan-Sala et al., blue; this paper, red). (**D**) The same UMAP colored by developmental timepoint. (**E**) Pairwise Sinkhorn similarity heatmap comparing replicates from this study (rows) to timepoints from Pijuan-Sala et al. (columns). (**F-H**) Analogous integration of the late timepoints from this study (E8.5-E10.0) with the reference dataset from Qiu et al., 2024[7]. (**F**) Joint UMAP colored by dataset. (**G**) The same UMAP colored by developmental timepoint. (**H**) Pairwise Sinkhorn similarity heatmap comparing replicates from this study (rows) to timepoints from Qiu et al. (columns). (**I**) Proportion of tracing donor and non-tracing host cells across all 16 embryos (mean 85%, range 52-99%). (**J**) UMAP of all cells colored by donor (blue) versus host (salmon) cell identity, excluding primitive erythrocytes. (**K**) UMAP colored by developmental domain. (**L**) Relative contributions of donor and host cells to each developmental domain across all embryos.

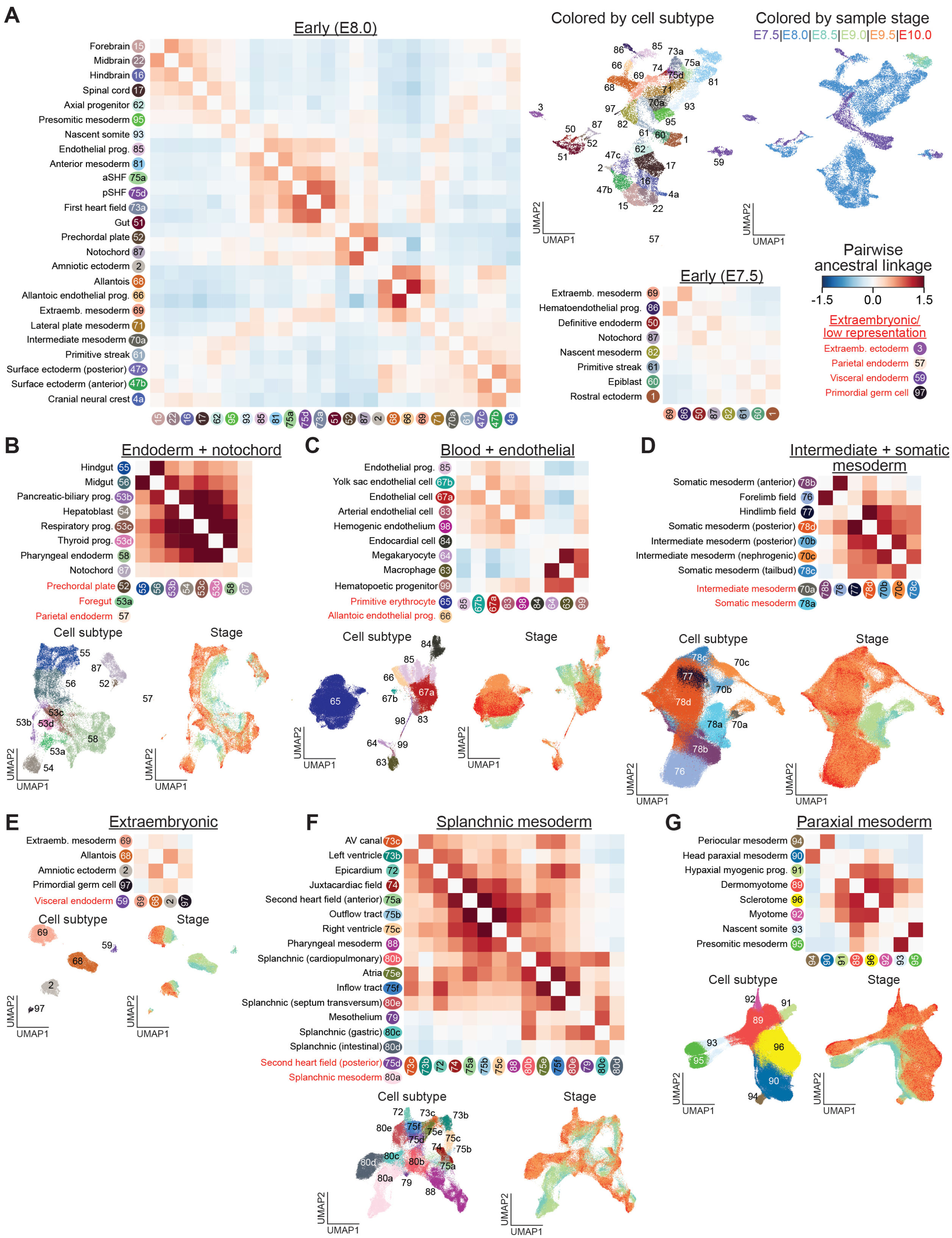
A
Early (E8.0)
Forebrain 15
Midbrain 22
Hindbrain 16
Spinal cord 17
Axial progenitor 62
Presomitic mesoderm 95
Nascent somite 93
Endothelial prog. 85
Anterior mesoderm 81
aSHF 75a
pSHF 75d
First heart field 73a
Gut 51
Prechordal plate 52
Notochord 87
Amniotic ectoderm 2
Allantois 68
Allantoic endothelial prog. 66
Extraemb. mesoderm 69
Lateral plate mesoderm 71
Intermediate mesoderm 70a
Primitive streak 61
Surface ectoderm (posterior) 47c
Surface ectoderm (anterior) 47b
Cranial neural crest 4a
Colored by cell subtype
Colored by sample stage
E7.5|E8.0|E8.5|E9.0|E9.5|E10.0
UMAP1
UMAP2
Early (E7.5)
Extraemb. mesoderm 69
Hematoendothelial prog. 86
Definitive endoderm 50
Notochord 87
Nascent mesoderm 82
Primitive streak 61
Epiblast 60
Rostral ectoderm 1
Pairwise ancestral linkage
-1.5
0.0
1.5
Extraembryonic/ low representation
Extraemb. ectoderm 3
Parietal endoderm 57
Visceral endoderm 59
Primordial germ cell 97
B
Endoderm + notochord
Hindgut 55
Midgut 56
Pancreatic-biliary prog. 53b
Hepatoblast 54
Respiratory prog. 53c
Thyroid prog. 53d
Pharyngeal endoderm 58
Notochord 87
Prechordal plate 52
Foregut 53a
Parietal endoderm 57
Cell subtype
Stage
C
Blood + endothelial
Endothelial prog. 85
Yolk sac endothelial cell 67b
Endothelial cell 67a
Arterial endothelial cell 83
Hemogenic endothelium 98
Endocardial cell 84
Megakaryocyte 64
Macrophage 63
Hematopoetic progenitor 99
Primitive erythrocyte 65
Allantoic endothelial prog. 66
Cell subtype
Stage
D
Intermediate + somatic mesoderm
Somatic mesoderm (anterior) 78b
Forelimb field 76
Hindlimb field 77
Somatic mesoderm (posterior) 78d
Intermediate mesoderm (posterior) 70b
Intermediate mesoderm (nephrogenic) 70c
Somatic mesoderm (tailbud) 78c
Intermediate mesoderm 70a
Somatic mesoderm 78a
Cell subtype
Stage
E
Extraembryonic
Extraemb. mesoderm 69
Allantois 68
Amniotic ectoderm 2
Primordial germ cell 97
Visceral endoderm 59
Cell subtype
Stage
F
Splanchnic mesoderm
AV canal 73c
Left ventricle 73b
Epicardium 72
Juxtacardiac field 74
Second heart field (anterior) 75a
Outflow tract 75b
Right ventricle 75c
Pharyngeal mesoderm 88
Splanchnic (cardiopulmonary) 80b
Atria 75e
Inflow tract 75f
Splanchnic (septum transversum) 80e
Mesothelium 79
Splanchnic (gastric) 80c
Splanchnic (intestinal) 80d
Second heart field (posterior) 75d
Splanchnic mesoderm 80a
Cell subtype
Stage
G
Paraxial mesoderm
Periocular mesoderm 94
Head paraxial mesoderm 90
Hypaxial myogenic prog. 91
Dermomyotome 89
Sclerotome 96
Myotome 92
Nascent somite 93
Presomitic mesoderm 95
Cell subtype
Stage

Figure S5. Cell subtype ancestral linkage and embryonic staging, related to Figure 2.

Ancestral linkage heatmaps with cell subtype labels and UMAP embeddings colored by cell subtype and sample stage for the following developmental domains: (**A**) Early embryonic timepoints (E7.5 and E8.0), (**B**) endoderm and notochord, (**C**) blood and endothelial, (**D**) intermediate and somatic mesoderm, (**E**) extraembryonic, (**F**) splanchnic mesoderm, and (**G**) paraxial mesoderm. Cell subtypes included in UMAPs but excluded from the ancestral linkage heatmaps due to low representation are colored in red.

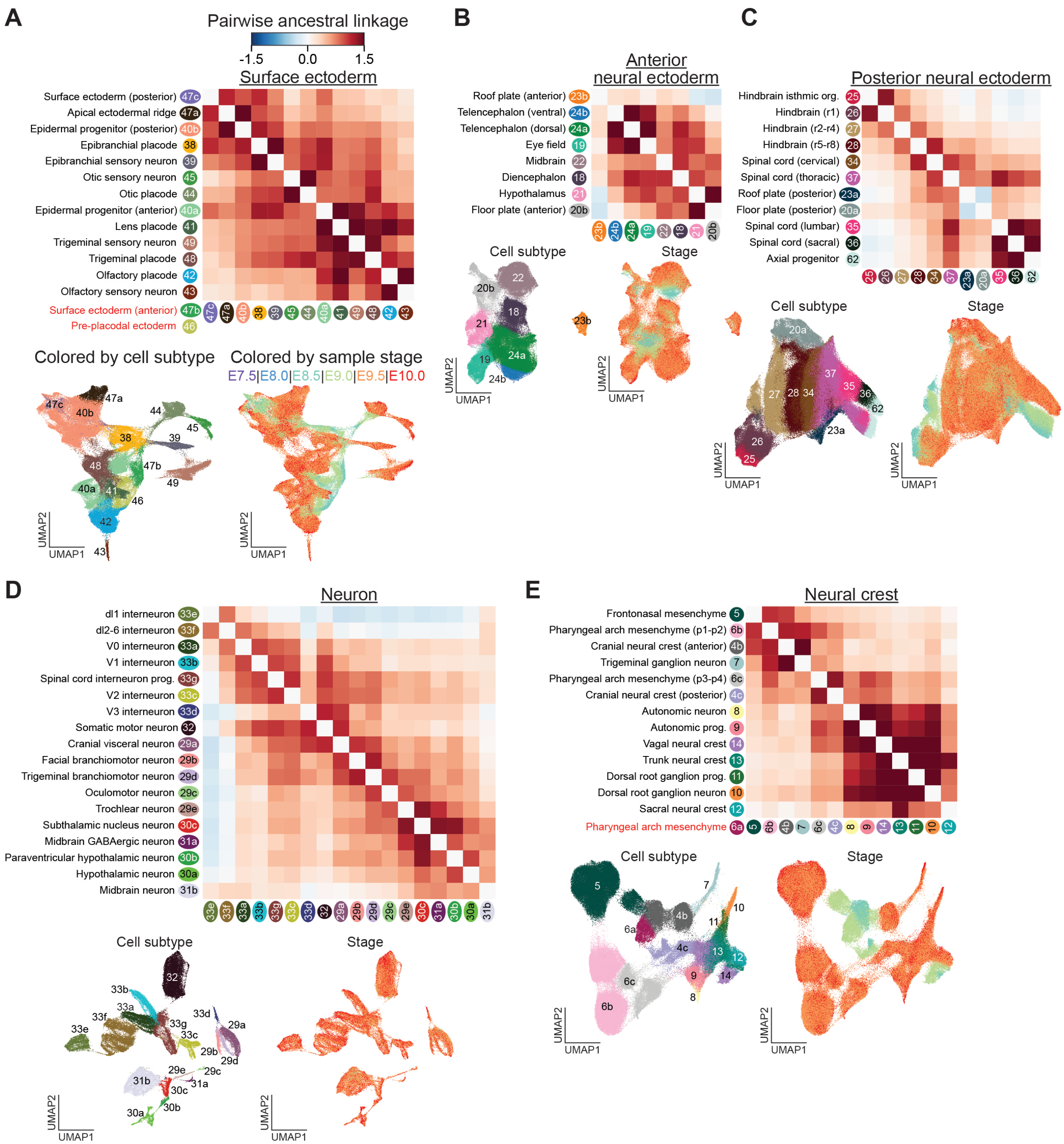

Figure S6. Cell subtype ancestral linkage and embryonic staging, related to Figure 2.

Ancestral linkage heatmaps with cell subtype labels and UMAP embeddings colored by cell subtype and sample stage for the following developmental domains: (**A**) surface ectoderm, (**B**) anterior neural ectoderm, (**C**) posterior neural ectoderm, (**D**) neurons, and (**E**) neural crest. Cell subtypes included in UMAPs but excluded from the ancestral linkage heatmaps due to low representation are colored in red.

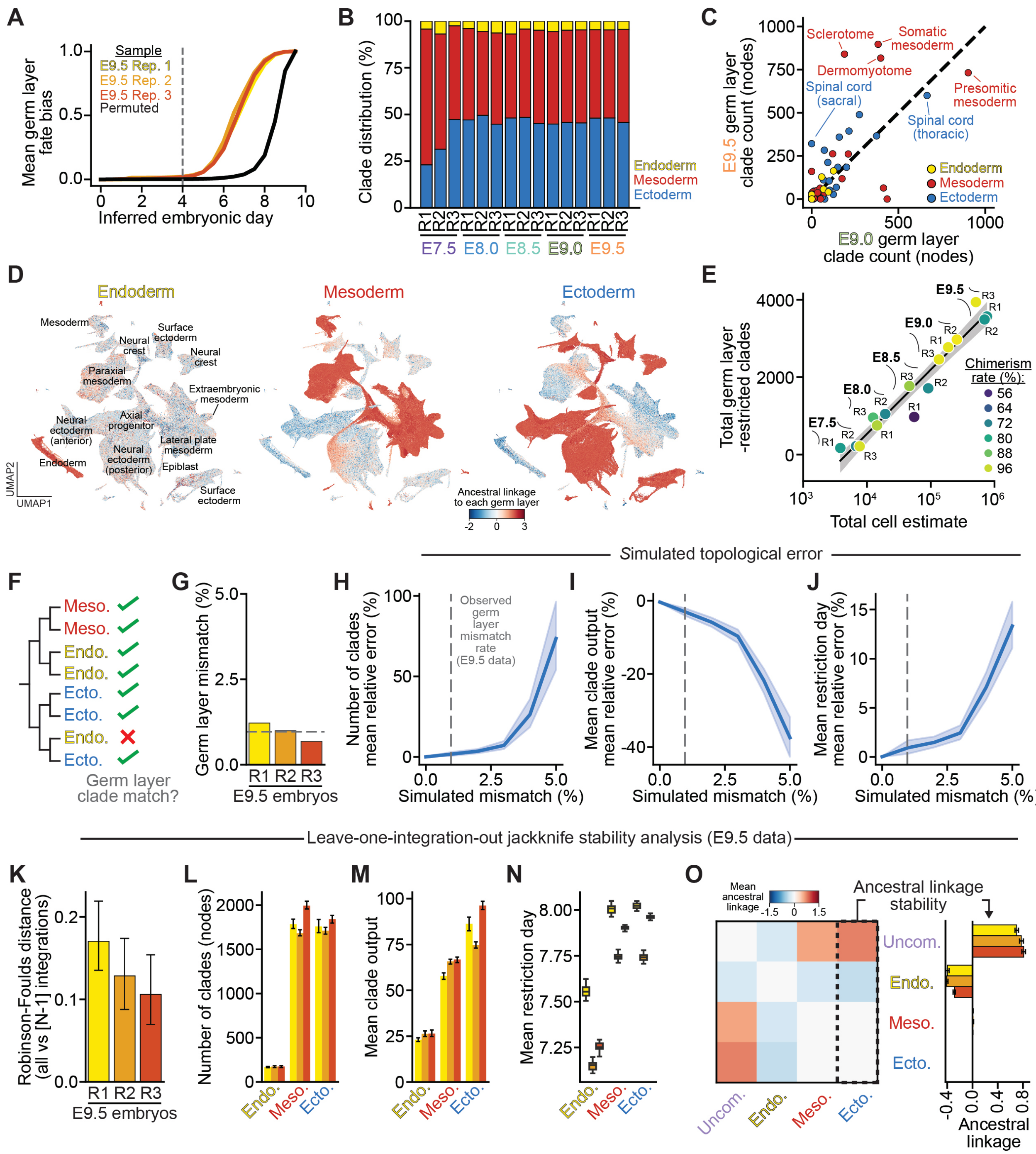

Figure S7. Quantification of germ layer specification dynamics with stability analysis, related to Figure 3.

(**A**) Mean germ layer-level fate bias over inferred embryonic day for three E9.5 replicates and a permuted control (black). The dashed line marks the earliest time at which fate bias is statistically significant (permutation test) across all three replicates. (**B**) Distribution of germ layer-restricted clades for each replicate (R1–R3) at each sampled timepoint (E7.5–E9.5), shown as the percentage of clades assigned to endoderm (yellow), mesoderm (red), or ectoderm (blue). (**C**) Number of germ layer-restricted clades contributing to each cell type at E9.0 vs. E9.5. Cell types are colored by germ layer assignment. (**D**) UMAP of all lineage traced cells colored by ancestral linkage to each germ layer (endoderm, mesoderm, ectoderm). Red indicates linkages that are closer than would be expected by chance and blue indicates the

opposite. (**E**) Total number of germ layer-restricted clades versus estimated total cell number for each embryo, labeled by timepoint (E7.5–E9.5) and replicate (R1–R3) and colored by chimerism rate. Line and shading indicate the linear fit and 95% CI. (**F**) Schematic of the percent germ layer mismatch metric: the germ layer label of each leaf is compared to the most parsimonious (Fitch-Hartigan) label for its parent. (**G**) Observed germ layer mismatch rate for E9.5 replicates R1-R3; dashed line indicates the mean (0.97% of cells). (**H-J**) Mean relative error in (**H**) the number of fate-restricted clades, (**I**) mean clade output, and (**J**) mean germ layer restriction day as a function of simulated topological error (germ layer mismatch rate, 0-5%). Line and shading indicate mean and 95% CI; vertical dashed line marks the observed E9.5 mismatch rate (**G**). (**K-O**) Leave-one-integration-out jackknife analysis (E9.5 data): trees were reconstructed after removing each intBC in turn (N-1 integrations) and compared to the full-data tree. (**K**) Normalized Robinson-Foulds (RF) distance between full and N-1 jackknife trees for R1-R3; mean ± 95% CI. (**L**) Number of fate-restricted clades, (**M**) mean clade output, and (**N**) mean restriction day for endoderm (Endo.), mesoderm (Meso.), and ectoderm (Ecto.), colored by replicate (R1-R3). (**O**) Left, mean pairwise ancestral linkage between uncommitted progenitors (Uncom.), Endo., Meso., and Ecto. across jackknife trees. Right, linkage of each population to ectoderm (boxed column) across jackknife trees (mean ± 95% CI), showing stability to integration dropout.

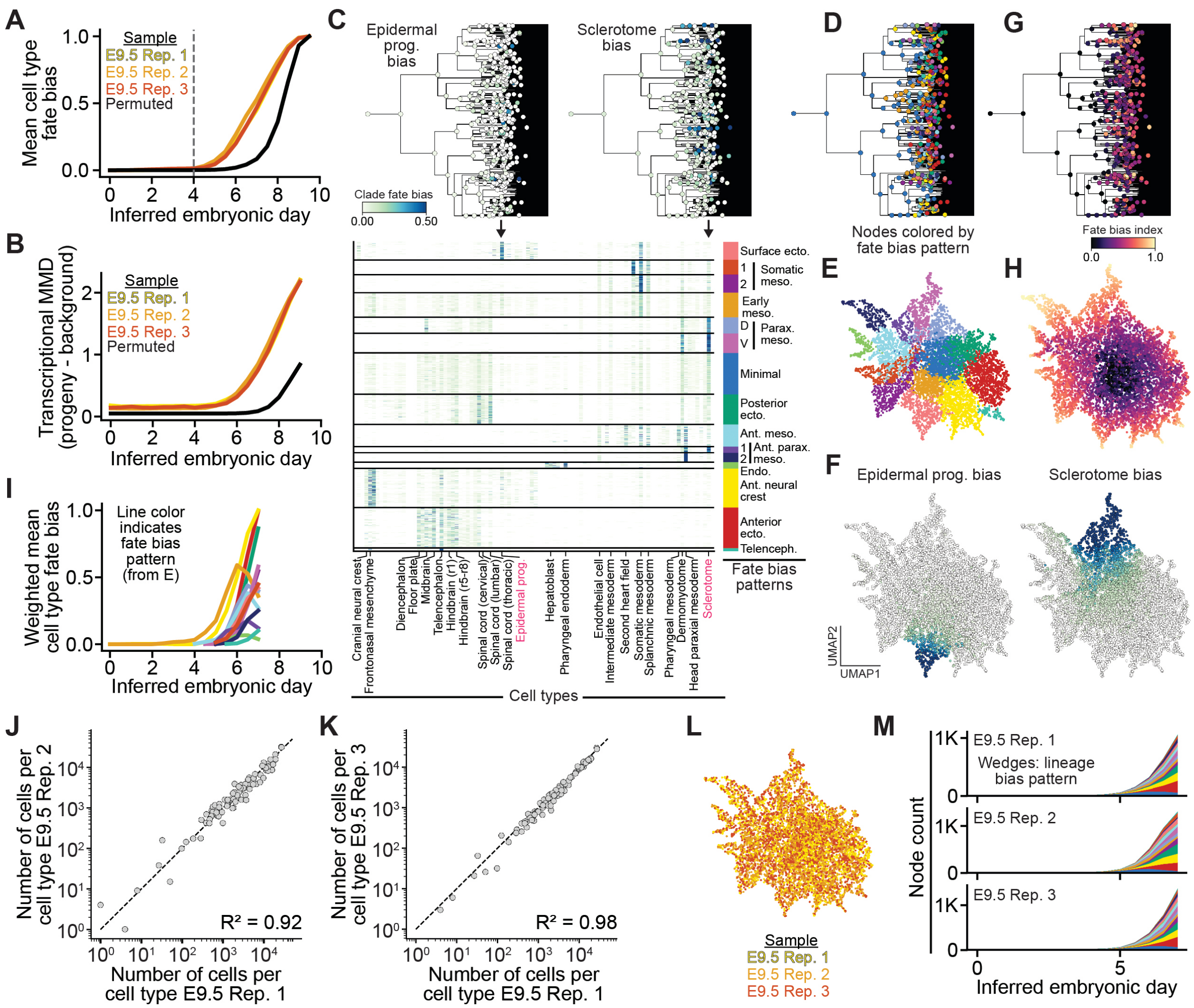

Figure S8. Quantification of fate bias and reproducibility across cell types, related to Figure 3.

(**A**) Mean cell type-level fate bias over inferred embryonic day for three E9.5 replicates and a permuted control (black). The dashed line marks the earliest time at which fate bias is statistically significant (permutation test) across all three replicates. (**B**) Mean transcriptional MMD (maximum mean discrepancy) between descendants of internal nodes and the overall distribution, for three E9.5 replicates and a permuted control (black). (**C**) Top: Example lineage tree (E9.5-R1-C2) with early ancestral nodes (inferred day <7 and >50 descendants) from E9.5 replicates colored by fate bias toward epidermal progenitor (left) and sclerotome (right) identities. Bottom: Heatmap representing early ancestral node fate bias (rows) across cell types (columns), grouped by dominant fate bias pattern (colored labels, right). (**D**) Example lineage tree with ancestral nodes colored by assigned fate bias pattern. (**E**) UMAP embedding of ancestral node fate distributions colored by assigned fate bias pattern. (**F**) UMAP embedding from (E) colored by fate bias towards epidermal progenitor (left) and sclerotome (right) identities as in (C). (**G,H**) Example tree and UMAP from (D,E) colored by cell type-level fate bias index. (**I**) Mean cell type-level fate bias for each lineage pattern weighted by abundance, with lines colored by fate bias pattern (from C). (**J, K**) Cell type abundances between E9.5 replicate 1 and replicate 2 (J, R^2= 0.92) or replicate 3 (K, R^2= 0.98). Each point represents a cell type. (**L**) UMAP embedding of ancestral nodes from (E) colored by E9.5 replicate. (**M**) Number of extant ancestral nodes over inferred embryonic day for each E9.5 replicate colored by fate bias patterns (from C).

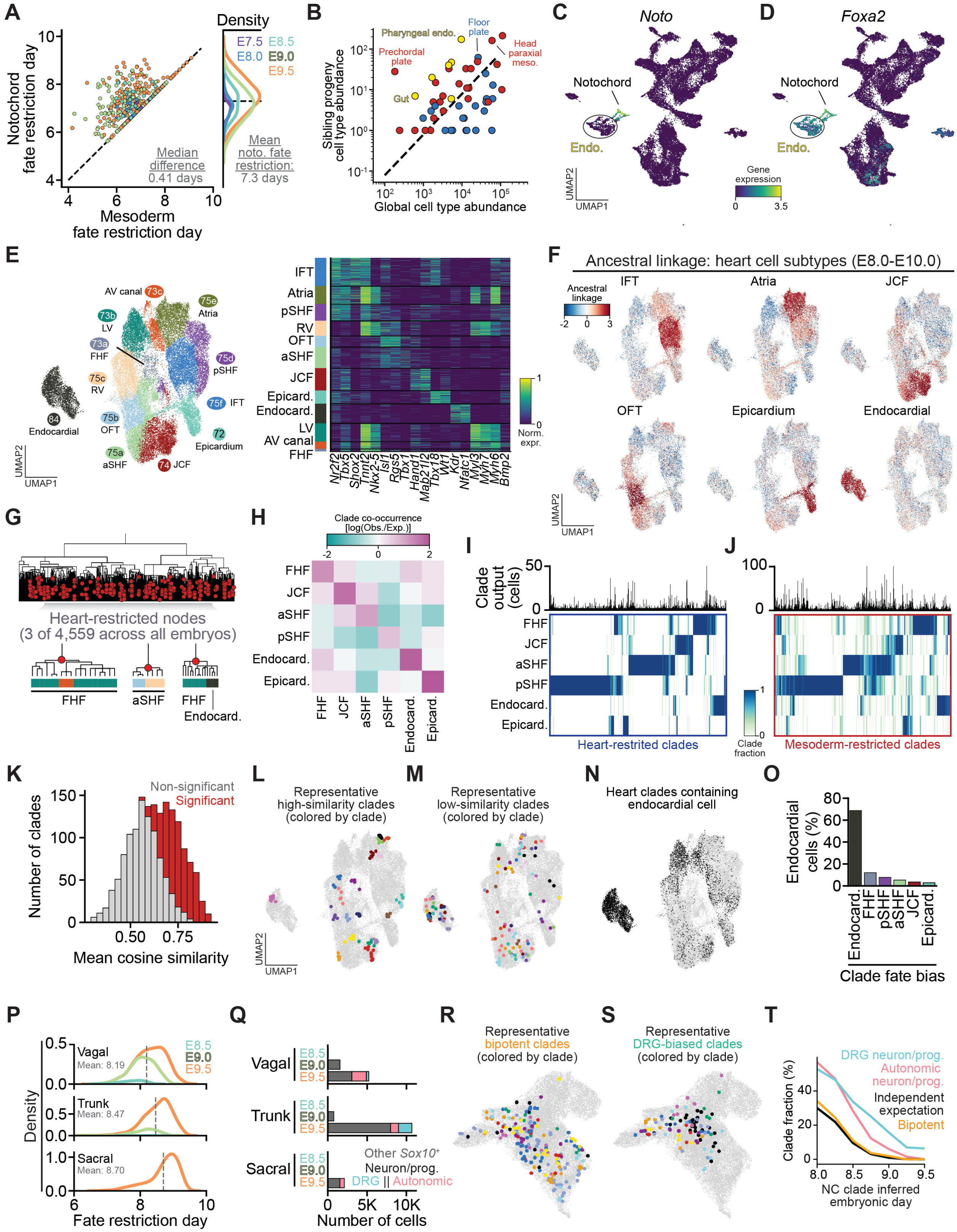

A
Density
E7.5 E8.5
E8.0 E9.0
E9.5
Notochord fate restriction day
Mesoderm fate restriction day
Median difference 0.41 days
Mean noto. fate restriction: 7.3 days
B
Pharyngeal endo.
Floor plate
Head paraxial meso.
Prechordal plate
Gut
Sibling progeny cell type abundance
Global cell type abundance
C
Noto
Notochord
Endo.
UMAP2
UMAP1
D
Foxa2
Notochord
Endo.
Gene expression
E
AV canal
Atria
LV
FHF
pSHF
RV
IFT
OFT
Endocardial
Epicardium
aSHF
JCF
IFT
Atria
pSHF
RV
OFT
aSHF
JCF
Epicard.
Endocard.
LV
AV canal
FHF
Norm. expr.
F
Ancestral linkage: heart cell subtypes (E8.0-E10.0)
Ancestral linkage
IFT
Atria
JCF
OFT
Epicardium
Endocardial
G
Heart-restricted nodes
(3 of 4,559 across all embryos)
FHF
aSHF
FHF
Endocard.
H
Clade co-occurrence [log(Obs./Exp.)]
FHF
JCF
aSHF
pSHF
Endocard.
Epicard.
I
Clade output (cells)
FHF
JCF
aSHF
pSHF
Endocard.
Epicard.
Heart-restrited clades
Clade fraction
J
Mesoderm-restricted clades
K
Non-significant
Significant
Number of clades
Mean cosine similarity
L
Representative high-similarity clades (colored by clade)
M
Representative low-similarity clades (colored by clade)
N
Heart clades containing endocardial cell
O
Endocardial cells (%)
Endocard.
FHF
pSHF
aSHF
JCF
Epicard.
Clade fate bias
P
Vagal
Mean: 8.19
Trunk
Mean: 8.47
Sacral
Mean: 8.70
E8.5
E9.0
E9.5
Density
Fate restriction day
Q
Vagal
Trunk
Sacral
Other Sox10+
Neuron/prog.
DRG || Autonomic
Number of cells
R
Representative bipotent clades (colored by clade)
S
Representative DRG-biased clades (colored by clade)
T
DRG neuron/prog.
Autonomic neuron/prog.
Independent expectation
Bipotent
Clade fraction (%)
NC clade inferred embryonic day

Figure S9. Supporting analyses for notochord and heart field fate restriction dynamics, related to Figure 4.

(**A-D**) Notochord: (**A**) Left: Scatter plot of mesoderm fate restriction day versus notochord fate restriction day for each notochord-restricted clade. Points are colored by each clade's sampled timepoint and points on the diagonal indicate concurrent restriction; the median difference between mesoderm and notochord restriction is 0.41 days. Right: Distribution of inferred notochord fate restriction times across triplicate sampled timepoints (E7.5–E9.5), shown as a kernel density estimate over clades in which the y-axis is probability density (area under curve = 1). Dashed line indicates the mean at inferred embryonic day 7.3. Note that we do not distinguish the Node from Notochord due to their high transcriptional similarity and differential timing. (**B**) Sibling clade cell type abundance versus global cell type abundance. Points above the diagonal are enriched among notochord sibling clades; selected cell types are labeled. Colors indicate germ layer. (**C**) UMAP of E7.5 and E8.0 cells colored by *Noto* expression, and (**D**) *Foxa2* expression. Notochord and endoderm populations are indicated**.** (**E-O**) Heart fields: (**E**) Left: UMAP of 12 annotated cardiac cell subtypes (as in Figure 4F). Right: heatmap of normalized expression of marker genes used for cardiac cell type annotation. IFT = inflow tract, pSHF = posterior second heart field, RV = right ventricle, OFT = outflow tract, aSHF = anterior SHF, JCF = juxtacardiac field, Epicard. = epicardial cells, Endocard. = endocardium, LV = left ventricle, AV canal = atrioventricular canal, FHF = first heart field. (**F)** Cardiac UMAPs colored by ancestral linkage to each cardiac cell type across E8.0-E10.0 embryos. (**G**) Example lineage tree (E9.5-R2-C1) with heart-restricted nodes (red circles) marked. Inset trees depict 3 of 4,559 heart-restricted nodes across all embryos. Color bars indicate cell type identity of descendant cells. (**H**) Heart field co-occurrence [log(observed/expected)] within heart-restricted clades across sampled timepoints (E8.0-E9.5). (**I**) Clustered heatmap of heart field (rows) representation for individual heart-restricted clades (columns). Clade output (number of cells) shown above. (**J**) Same as (I) for individual mesoderm-restricted clades (columns). (**K**) Histogram of mean cosine similarity of scVI embedding of heart cells within heart-restricted clades across E8.0-E10.0 embryos**.** Clades with statistically significant within-clade similarity are highlighted in red (permutation test). (**L**) Representative high-similarity and (**M**) low-similarity heart-restricted clades on the UMAP from (E). 10 colors indicate 10 randomly sampled clades. (**N**) Heart-restricted clades containing at least one endocardial cell (black) on the UMAP. (**O**) Distribution of fate bias assignments for endocardial cell-generating heart-restricted clades. (**P**) Distribution of inferred vagal, trunk, and sacral NC fate restriction timing across sampled timepoints (E8.5-E9.5). Dashed line indicates the mean. (**Q**) Cell type composition of vagal, trunk, and sacral NC clades across sampled timepoints, colored by DRG neuron/progenitor (teal), autonomic neuron/progenitor (pink), and other *Sox10*$^{+}$ cells (gray). UMAP of posterior NC cells colored by (**R**) representative bipotent and (**S**) DRG-biased trunk NC clades. 10 colors indicate 10 randomly sampled clades. (**T**) Probability of an E9.5 clade containing DRG, autonomic, and bipotent outputs as a function of NC-restricted clade depth (inferred embryonic day). Black line indicates the expected bipotent frequency under statistical independence between DRG and autonomic fate occurrence.

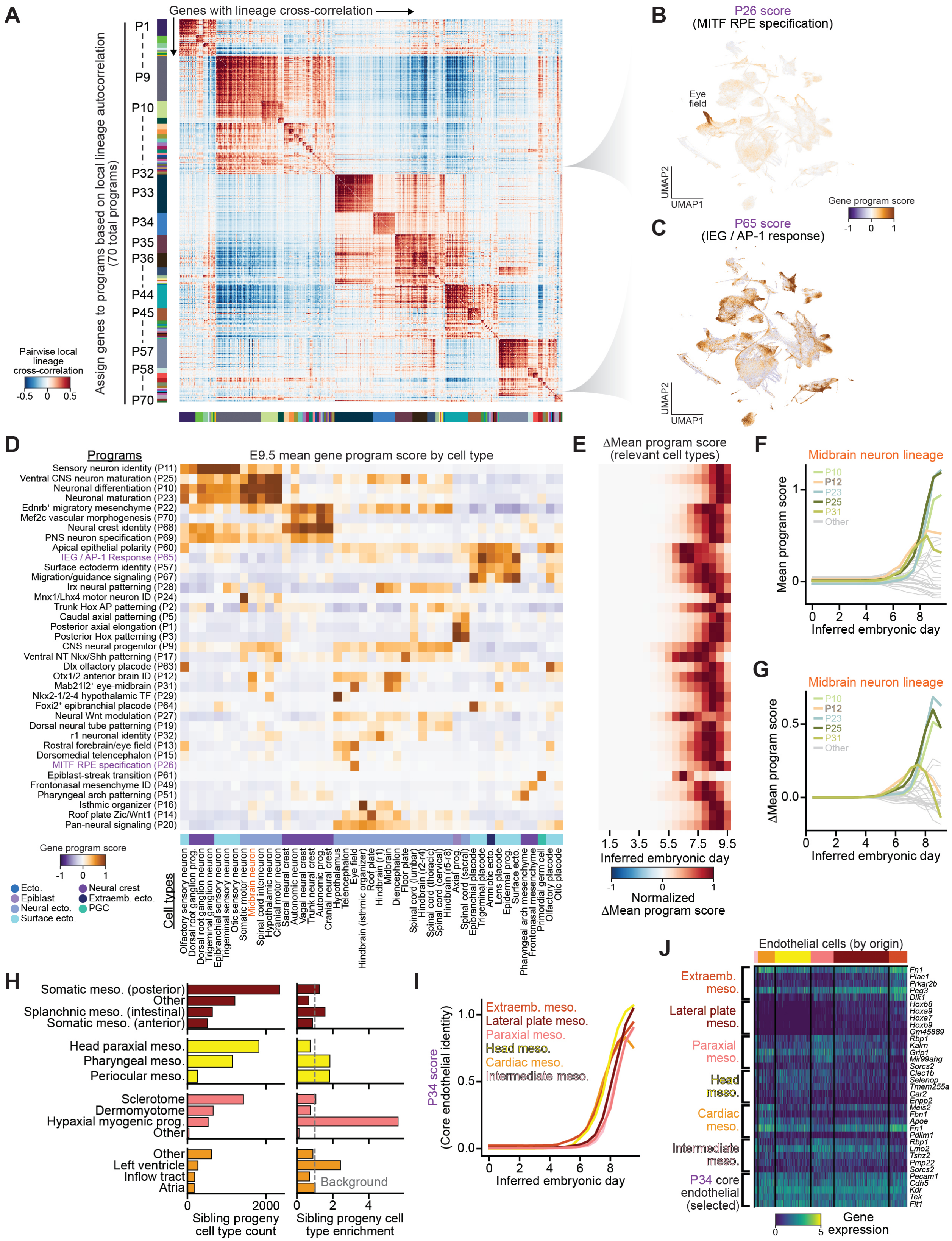
A
Genes with lineage cross-correlation
Assign genes to programs based on local lineage autocorrelation (70 total programs)
P1
P9
P10
P32
P33
P34
P35
P36
P44
P45
P57
P58
P70
Pairwise local lineage cross-correlation
-0.5 0 0.5
B
P26 score
(MITF RPE specification)
Eye field
UMAP2
UMAP1
Gene program score
-1 0 1
C
P65 score
(IEG / AP-1 response)
UMAP2
UMAP1
D
Programs
E9.5 mean gene program score by cell type
Sensory neuron identity (P11)
Ventral CNS neuron maturation (P25)
Neuronal differentiation (P10)
Neuronal maturation (P23)
Ednrb⁺ migratory mesenchyme (P22)
Mef2c vascular morphogenesis (P70)
Neural crest identity (P68)
PNS neuron specification (P69)
Apical epithelial polarity (P60)
IEG / AP-1 Response (P65)
Surface ectoderm identity (P57)
Migration/guidance signaling (P67)
Irx neural patterning (P28)
Mnx1/Lhx4 motor neuron ID (P24)
Trunk Hox AP patterning (P2)
Caudal axial patterning (P5)
Posterior axial elongation (P1)
Posterior Hox patterning (P3)
CNS neural progenitor (P9)
Ventral NT Nkx/Shh patterning (P17)
Dlx olfactory placode (P63)
Otx1/2 anterior brain ID (P12)
Mab21l2⁺ eye-midbrain (P31)
Nkx2-1/2-4 hypothalamic TF (P29)
Foxi2⁺ epibranchial placode (P64)
Neural Wnt modulation (P27)
Dorsal neural tube patterning (P19)
r1 neuronal identity (P32)
Rostral forebrain/eye field (P13)
Dorsomedial telencephalon (P15)
MITF RPE specification (P26)
Epiblast-streak transition (P61)
Frontonasal mesenchyme ID (P49)
Pharyngeal arch patterning (P51)
Isthmic organizer (P16)
Roof plate Zic/Wnt1 (P14)
Pan-neural signaling (P20)
Gene program score
-1 0 1
Ecto.
Epiblast
Neural ecto.
Surface ecto.
Neural crest
Extraemb. ecto.
PGC
Cell types
Olfactory sensory neuron
Dorsal root ganglion prog.
Dorsal root ganglion neuron
Trigeminal ganglion neuron
Epibranchial sensory neuron
Trigeminal sensory neuron
Otic sensory neuron
Somatic motor neuron
Midbrain neuron
Spinal cord interneuron
Hypothalamic neuron
Cranial motor neuron
Sacral neural crest
Autonomic neuron
Vagal neural crest
Trunk neural crest
Autonomic prog.
Cranial neural crest
Hypothalamus
Telencephalon
Eye field
Hindbrain (isthmic organizer)
Roof plate
Hindbrain (r1)
Midbrain
Diencephalon
Floor plate
Spinal cord (lumbar)
Hindbrain (r2-r4)
Spinal cord (thoracic)
Spinal cord (cervical)
Hindbrain (r5-r8)
Axial prog.
Spinal cord (sacral)
Epibranchial placode
Trigeminal placode
Amniotic ecto.
Lens placode
Epidermal prog.
Surface ecto.
Pharyngeal arch mesenchyme
Frontonasal mesenchyme
Primordial germ cell
Olfactory placode
Otic placode
E
ΔMean program score
(relevant cell types)
1.5 3.5 5.5 7.5 9.5
Inferred embryonic day
-1 0 1
Normalized
ΔMean program score
F
Midbrain neuron lineage
P10
P12
P23
P25
P31
Other
Mean program score
0
1
0 2 4 6 8
Inferred embryonic day
G
Midbrain neuron lineage
P10
P12
P23
P25
P31
Other
ΔMean program score
0.0
0.5
0 2 4 6 8
Inferred embryonic day
H
Somatic meso. (posterior)
Other
Splanchnic meso. (intestinal)
Somatic meso. (anterior)
Head paraxial meso.
Pharyngeal meso.
Periocular meso.
Sclerotome
Dermomyotome
Hypaxial myogenic prog.
Other
Other
Left ventricle
Inflow tract
Atria
0 2000
Sibling progeny cell type count
0 2 4
Background
Sibling progeny cell type enrichment
I
P34 score
(Core endothelial identity)
0.0
0.5
1.0
Extraemb. meso.
Lateral plate meso.
Paraxial meso.
Head meso.
Cardiac meso.
Intermediate meso.
0 2 4 6 8
Inferred embryonic day
J
Endothelial cells (by origin)
Extraemb. meso.
Lateral plate meso.
Paraxial meso.
Head meso.
Cardiac meso.
Intermediate meso.
P34 core endothelial (selected)
Fn1
Plac1
Prkar2b
Peg3
Dlk1
Hoxb8
Hoxa9
Hoxa7
Hoxb9
Gm45889
Rbp1
Kalrn
Grip1
Mir99ahg
Sorcs2
Clec1b
Selenop
Tmem255a
Car2
Enpp2
Meis2
Fbn1
Apoe
Fn1
Pdlim1
Rbp1
Lmo2
Tshz2
Pmp22
Sorcs2
Pecam1
Cdh5
Kdr
Tek
Flt1
0 5
Gene expression

Figure S10. Gene regulatory program nomination and activity across ectodermal cell types, related to Figure 5.

(**A**) Pairwise local lineage cross-correlation matrix for 1,322 genes assigned to programs across all embryos. Genes are ordered by their hierarchical clustering assignment via Hotspot, revealing blocks of coordinated structure. Colored bars indicate assignment of genes to individual gene programs (P1-P70). (**B**) UMAP of all sampled cells colored by P26 (MITF RPE specification) or (**C**) P65 (IEG/AP-1 response) program scores. Cell type-specific activation of P26 in the eye field is annotated in (B). For a full list of gene regulatory program names, abbreviations, and descriptions of their assignment see **Table S5**. (**D**) Mean score for each program (rows) across ectodermal cell types (columns) in E9.5 embryos. Cell type developmental domain assignments are indicated by colored bars along the bottom. Highlighted programs in purple: P65 (IEG/AP-1 response) and P26 (MITF RPE specification) from (B,C). Highlighted cell type in orange is midbrain neuron. (**E**) Change in mean program score (ΔMean program score) over inferred embryonic day in E9.5 lineage trees for relevant cell types (mean >0.5 in at least one timepoint), normalized per program. (**F**) Mean program score and (**G**) change in mean program score over inferred embryonic day for gene programs within the midbrain neuron lineage. Programs include P10 (neuronal differentiation), P12 (Otx1/2 anterior brain ID), P23 (neuronal maturation), P25 (ventral CNS neuron maturation), and P31 (Mab21l2^{+} eye-midbrain). (**H**) Endothelial-restricted clade sibling progeny counts (left) and fold enrichment relative to background (right) for E9.5 replicates 1-3. (**I**) P34 (core endothelial identity) mean program score over inferred embryonic day in E9.5 lineage trees colored by inferred endothelial origin. (**J**) Heatmap of E9.5 endothelial cells grouped by inferred origin showing the expression of top 5 differentially expressed genes for each origin as well as selected genes from core endothelial program.

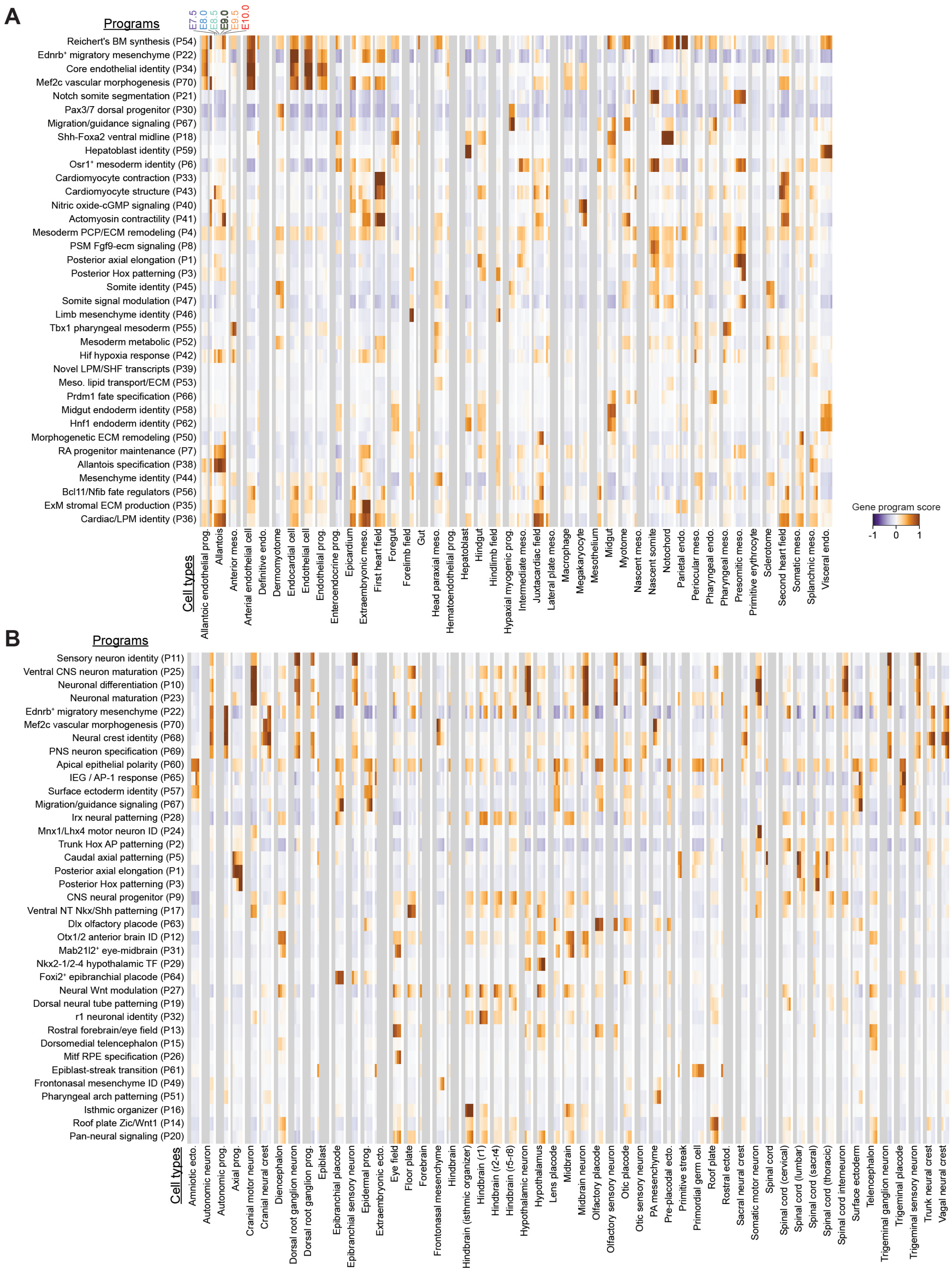

Figure S11. Gene program activity across cell types over time, related to Figure 5.

Gene program activity (rows) across sampled timepoints from E7.5 to E10.0 for (**A**) mesodermal and endodermal as well as (**B**) ectodermal cell types. Timepoints where cell types are not present are shown in grey.

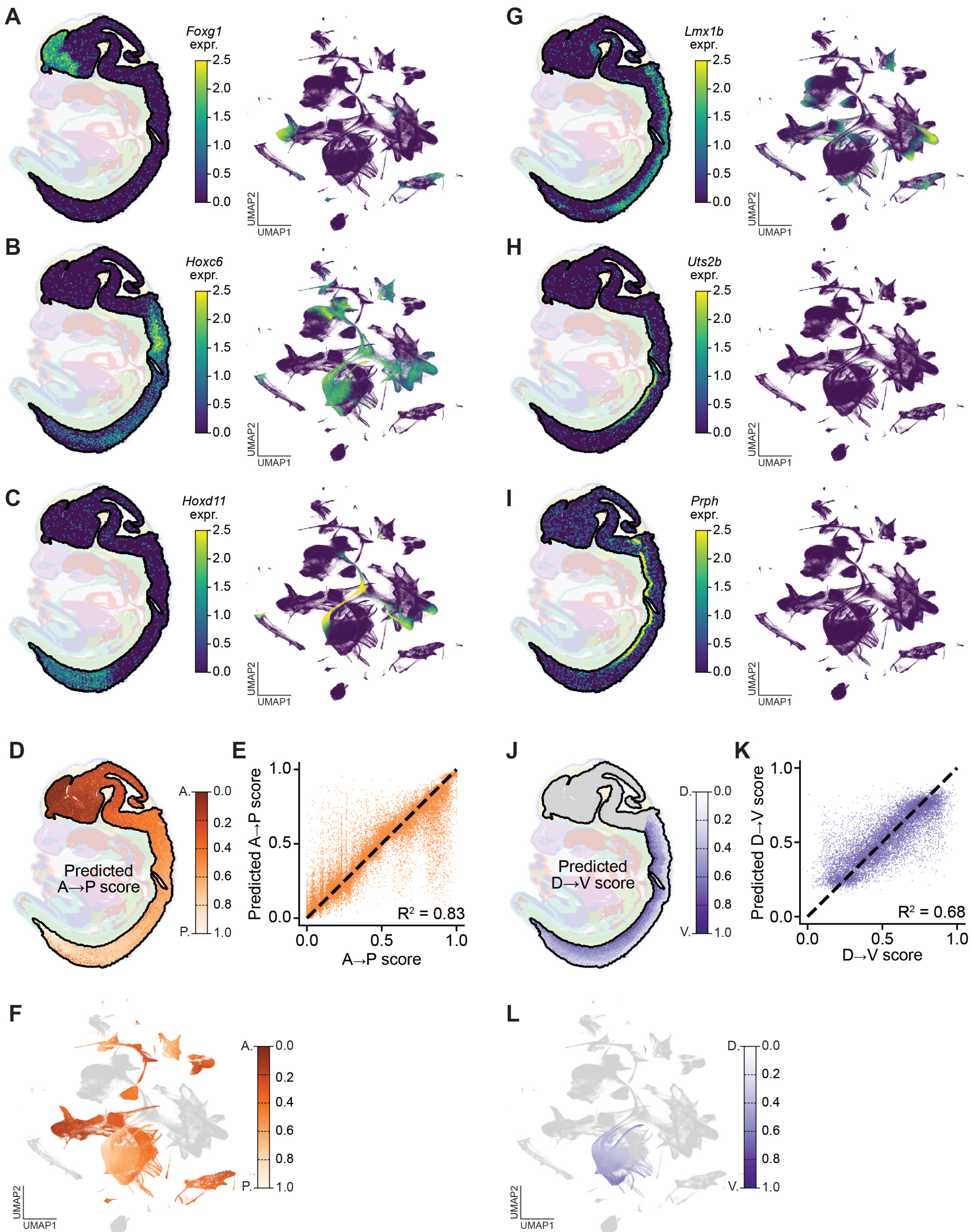

A
Foxg1 expr.
2.5
2.0
1.5
1.0
0.5
0.0
UMAP2
UMAP1
B
Hoxc6 expr.
C
Hoxd11 expr.
D
Predicted A→P score
A.
P.
0.0
0.2
0.4
0.6
0.8
1.0
E
Predicted A→P score
A→P score
R² = 0.83
F
G
Lmx1b expr.
H
Uts2b expr.
I
Prph expr.
J
Predicted D→V score
D.
V.
K
Predicted D→V score
D→V score
R² = 0.68
L

Figure S12. Spatial transcriptomics-based inference of spatial axes, related to Figure 6.

(**A-C**) Left: spatial transcriptomics data from a sagittal section of a E13.5 mouse embryo (Stereo-seq; Chen et al., 2022). Neural ectoderm is outlined and colored by expression of example anterior-posterior (A-P) patterning genes, while other tissues are shown in the background for reference. Right: UMAP embedding of all cells in our dataset colored by the expression of the same genes: (**A**) *Foxg1*, (**B**) *Hoxc6*, and (**C**) *Hoxd11*. (**D**) Predicted A-P score of stereo-seq spots, using our model (5-fold cross validation). (**E**) Concordance of predicted and inferred A-P position across the stereo-seq dataset (R^2= 0.83). (**F**) UMAP embedding of all cells in our dataset colored by predicted A-P score. (**G-I**) Same as (A-C) for dorsal-ventral (D-V) patterning genes: (**G**) *Lmx1b*, (**H**) *Uts2b*, and (**I**) *Prph*. (**J,K**) Same as (D,E) for D-V score. (**J**) Predicted D-V score of stereo-seq spots, using our model (5-fold cross validation) and (**K**) Concordance of predicted and inferred D-V position across the stereo-seq dataset (R^2= 0.68). (**L**) UMAP embedding of all cells in our dataset colored by predicted D-V score.

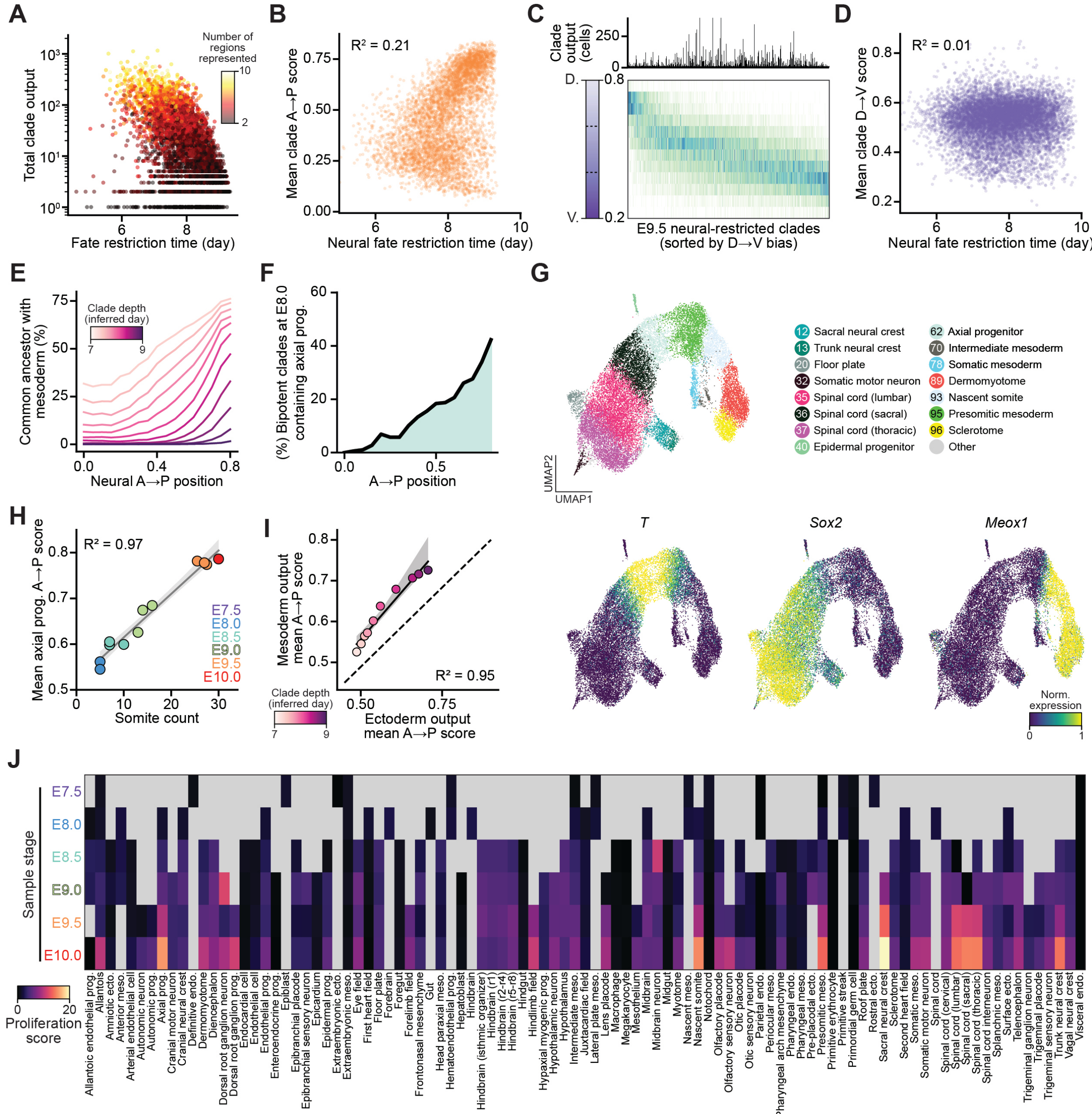

Figure S13. Supporting analysis for spatial axes, regionalization, and axis elongation, related to Figure 7.

(**A**) Total clade output (number of descendant cells) versus neural fate restriction time. Points are colored by the number of neural regions represented in each clade. (**B**) Mean A-P score of neural-restricted clades versus fate restriction time. (**C**) Heatmap showing the distribution of D-V positions (rows) for spinal cord clades in E9.5 embryos with >10 descendants (columns), ordered by mean D-V score. Clade output (number of cells) shown above. (**D**) Same as (B) but for mean D-V score of spinal cord clades. (**E**) Fraction of neural cells by A-P position sharing a common ancestor with the mesoderm at clade depths from E7.0-E9.0 (inferred embryonic day). (**F**) Fraction of bipotent clades with common ancestor at E8.0 that contain observed axial progenitors at E9.5, plotted across the A-P axis. (**G**) Top: UMAP of E9.5 posterior neural-restricted clades and their siblings colored by cell type (numbering as in Figure 2) and Bottom: *T*, *Sox2*, and *Meox1* expression. (**H**) Mean axial progenitor A-P score versus somite number across developmental timepoints. Trend line indicates linear regression fit (R^2= 0.97). (**I**) Mean A-P scores of mesodermal versus ectodermal outputs from bipotent clades at depths from E7.0-E9.0 (inferred embryonic day). Trend line

indicates linear regression fit (R^2= 0.95); dashed line indicates y = x. (**J**) Proliferation scores (calculated as mean number of cells sharing a common ancestor within the previous 24 hours) across cell types and developmental timepoints (E7.5-E10.0).

Supplemental References

1. Price, M.N., Dehal, P.S., and Arkin, A.P. (2010). FastTree 2--approximately maximum-likelihood trees for large alignments. PLoS One *5*, e9490. https://doi.org/10.1371/journal.pone.0009490.
2. Chu, G., Schmidt, H., and Raphael, B.J. (2026). LAML-Pro: Joint Maximum Likelihood Inference of Cell Genotypes and Cell Lineage Trees. https://doi.org/https://doi.org/10.1093/bioinformatics/btag219.
3. Jones, M.G., Khodaverdian, A., Quinn, J.J., Chan, M.M., Hussmann, J.A., Wang, R., Xu, C., Weissman, J.S., and Yosef, N. (2020). Inference of single-cell phylogenies from lineage tracing data using Cassiopeia. Genome Biol. *21*, 92. https://doi.org/10.1186/s13059-020-02000-8.
4. Prillo, S., Ravoor, A., Yosef, N., and Song, Y.S. (2026). ConvexML: Fast and accurate branch length estimation under irreversible mutation models, illustrated through applications to CRISPR/Cas9-based lineage tracing. Syst. Biol. *75*, 115–134. https://doi.org/https://doi.org/10.1093/sysbio/syaf054.
5. Bondarenko, V., Nikolaev, M., Kromm, D., Belousov, R., Wolny, A., Blotenburg, M., Zeller, P., Rezakhani, S., Hugger, J., Uhlmann, V., et al. (2023). Embryo-uterine interaction coordinates mouse embryogenesis during implantation. EMBO J. *42*, e113280. https://doi.org/10.15252/embj.2022113280.
6. Pijuan-Sala, B., Griffiths, J.A., Guibentif, C., Hiscock, T.W., Jawaid, W., Calero-Nieto, F.J., Mulas, C., Ibarra-Soria, X., Tyser, R.C.V., Ho, D.L.L., et al. (2019). A single-cell molecular map of mouse gastrulation and early organogenesis. Nature *566*, 490–495. https://doi.org/10.1038/s41586-019-0933-9.
7. Qiu, C., Martin, B.K., Welsh, I.C., Daza, R.M., Le, T.-M., Huang, X., Nichols, E.K., Taylor, M.L., Fulton, O., O'Day, D.R., et al. (2024). A single-cell time-lapse of mouse prenatal development from gastrula to birth. Nature *626*, 1084–1093. https://doi.org/10.1038/s41586-024-07069-w.
